# Obese Subjects without Eating Disorders Experience Binge Episodes Also Independently of Emotional Eating and Personality Traits among University Students of Southern Italy

**DOI:** 10.3390/brainsci11091145

**Published:** 2021-08-29

**Authors:** Ines Villano, Ciro Rosario Ilardi, Stefania Arena, Chiara Scuotto, Maria Gloria Gleijeses, Giovanni Messina, Antonietta Messina, Vincenzo Monda, Marcellino Monda, Alessandro Iavarone, Sergio Chieffi, Marco La Marra

**Affiliations:** 1 Department of Experimental Medicine, University of Campania “Luigi Vanvitelli”, 80138 Naples, Italy; gloria.gleijeses@gmail.com (M.G.G.); Antonietta.messina@unicampania.it (A.M.); vincenzo.monda@unicampania.it (V.M.); marcellino.monda@unicampania.it (M.M.); sergio.chieffi@unicampania.it (S.C.); marco.lamarra@unicampania.it (M.L.M.); 2 Department of Psychology, University of Campania “Luigi Vanvitelli”, 81100 Caserta, Italy; chiara.scuotto1@gmail.com; 3 Department of Advanced Medical and Surgical Sciences, University of Campania “Luigi Vanvitelli”, 80138 Naples, Italy; stefania.arena@unicampania.it; 4 Department of Clinical and Experimental Medicine, University of Foggia, 71122 Foggia, Italy; giovanni.messina@unifg.it; 5 Neurological Unit, CTO Hospital, AORN “Ospedali dei Colli”, 80131 Naples, Italy; aleiavarone@gmail.com

**Keywords:** emotional eating, personality, obesity, overeating, analysis of covariance

## Abstract

It is widely acknowledged that obesity is a growing public clinical issue involving both physical and psychological well-being. Nevertheless, the relationship between psychological features and weight gain is still unclear. Although emotional eating (EE) and personality traits are considered significant predictors of eating disorders, their role in obesity without eating disorders (OB-wed) is far from proven. The present study aimed at investigating the cumulative effect of EE and personality traits on overeating behavior in a sample of 266 university students (169 female; mean age = 21.85, SD = 2.39) stratified based on their body mass index (BMI; normal weight, overweight, obese). They were enrolled during free screening days promoted by the Human Dietetic and Sport Service of a Southern Italian university. The results show a psychological pattern of increasing overeating behavior and lower Self-Directedness combined with higher Sadness and Anger. However, OB-wed subjects overate regardless of this emotional/personological configuration.

## 1. Introduction

The World Health Organization formally recognized obesity as a growing public health problem in both developed and developing countries [1,2,3]. Obesity is a multifactorial clinical issue resulting from the interaction between genetic, physiological, and psychological factors [4,5]. Higher body weight represents a risk factor for several chronic diseases including type 2 diabetes, hypertension, myocardial infarction, dyslipidemias, vasculopathies, non-alcoholic liver steatosis, kidney disease, obstructive sleep apnea syndrome, and some forms of cancer; it also increases the mortality rate for all causes [6,7,8]. Although the negative association between obesity and health status is widely acknowledged, the relationship binding obesity to psychological well-being is unclear, and it is still a matter of debate [9].

In recent decades, clinical research has explored the relation between psychological features and obesity pathogenesis. In particular, several studies investigated the role of emotional eating (EE) [10] and personality traits [11,12].

According to the psychosomatic theory [13], EE is a feeding behavior characterized by the tendency to overeat in response to relevant emotional triggers [14]. Emotional eaters appear to be negative emotion-driven (e.g., anger, sadness, or boredom) [15,16], with overeating that mitigates emotional distress [17]. However, some studies suggested that also positive emotions may catalyze this behavioral pattern [18]. EE was related to high calorie intake, e.g., sugar or fat [19], higher body mass index (BMI) [20], poorer psychological well-being, and an increased rate of psychopathological disorders including depression, anxiety, apathy, obsessive-compulsive disorder, schizophrenia, and different types of eating disorders [21,22,23,24,25,26,27,28,29]. EE could be the result of an inverse relationship between higher body weight and the ability to cope with stress. This relationship may arise from a hypothalamic–pituitary–adrenal (HPA) axis dysregulation [30]. It is well known that the HPA axis represents a pivotal mediator of the stress response system in vertebrates, regulating homeostatic maintenance and behavioral responses to both internal and external threats [31]. 

EE is a high-risk behavior in eating disorders but can also be observed in overweight and/or obese individuals without eating disorders (OB-wed) [32]. Albeit some studies found a linear association between body weight and EE [28], the direction and extent of this association remain unclear. For instance, cross-sectional studies showed positive correlations [33,34], no correlation [35,36], and also negative correlations [37,38] between EE and weight gain. Accordingly, EE could also occur independently of eating attitudes. Indeed, psychological traits such as impulsivity [17], harm avoidance [39], reward dependence [40], susceptibility to hunger [41], neuroticism, and low conscientiousness [42] could independently predict overeating as well. These dimensions collectively outline part of the personality spectrum.

By definition, personality refers to a relatively stable pattern of psychological traits and underlies an individual’s tendency to think, act, and feel in a certain way. It was related to unhealthy behaviors [43], sedentary lifestyles, and high calorie intake [44]. Furthermore, it seems to affect weight gain through psychological mechanisms interacting with the ability to cope with stress [45]. By extension, personality plays a pivotal role in eating behavior [11,41]. Several studies attempting to delineate a prototypical personality pattern of individuals with OB-wed produced mixed results. Sensitivity to anxiety, extroversion, low consciousness, high impulsiveness, and low order appear to be the most representative personality traits [12,46,47,48]. However, the employment of multiple methodological approaches and different personality assessment tools does not allow drawing a clear prototypical personality profile of OB-wed subjects.

According to Cloninger’s psychobiological model [49,50], personality is composed of temperamental (i.e., Harm Avoidance, Novelty Seeking, Reward Dependence, and Persistence) and character (i.e., Self-Directedness, Cooperativeness, and Self-Transcendence) traits. Temperamental traits are inherited, manifest since childhood, and reflect the human tendency to respond to novelty, danger, or punishment; furthermore, they engage with memory and habit consolidation. On the other hand, character dimensions are partly inherited and partly experience-influenced; they refer to self-concept, and one’s goals and values. Each dimension is accounted for by unique genetic variance and is independent of sociocultural factors [50]. Both temperamental and character traits were operationalized through the Temperament and Character Inventory (TCI), a self-report questionnaire exploring features of personality according to Cloninger’s model [49,50]. Studies using the TCI for assessing personality in obese subjects produced heterogeneous results. In some studies, obese patients scored higher in Novelty Seeking and poorer in Reward Dependence, Persistence, Self-Directedness, and Self-Transcendence [11,39]. Differently, other studies found higher scores on Harm Avoidance [39,51] or Novelty Seeking [52], and lower scores on Persistence [51,52]. Interestingly, comparing obese groups with and without binge eating disorders (BEDs), the BED subjects showed lower Self-Directedness compared to no-BED subjects [25]. Conversely, both groups scored higher on Novelty Seeking and Harm Avoidance, and lower on Cooperativeness and Self-Directedness than normal-weight individuals [25]. Furthermore, different results were found between obese treatment seekers and non-seekers [48].

Although personality traits might contribute, in their pathological variants, to weight gain in clinical populations, we are far from detecting a structural personality pattern in essential obesity. Indeed, as previously outlined, no structural psychological configuration of OB-wed subjects can be currently supported.

Given the progressive growth of the overweight/obesity prevalence in recent decades, the relation between overeating and emotional and personality dimensions in influencing weight rise needs to be explored. On the one hand, this could increase the reliability of psychodiagnostic assessment tools; on the other hand, it could help to configure tailored weight loss treatments. In this vein, our study aimed at investigating the role of structural personality traits and EE in modulating overeating conducts in a BMI-ranked community sample including normal-weight, overweight, and obese individuals without eating disorders. We expected statistically significant differences between obese and normal-weight subjects.

## 2. Materials and Methods

### 2.1. Participants

This study was carried out on 266 participants (169 females and 97 males) aged between 18 and 29 years (mean age = 21.85; SD = 2.39). All participants were recruited at the Department of Experimental Medicine, Section of Human Physiology and Human Dietetic Service, on the free screening days intended for university students and promoted by the University of Campania “Luigi Vanvitelli”.

According to the Structured Clinical Interview (SCID) for DSM 5 [53], no participants met diagnostic criteria for Feeding and Eating Disorders (Anorexia nervosa, Bulimia nervosa, BED, Avoidant/restrictive food intake disorder, Other Specified Feeding or Eating Disorder, Unspecified Feeding and Eating Disorders). The inclusion/exclusion criteria were as follows: absence of intellectual or linguistic deficits, absence of neurological, psychiatric, or psychopathological disorders (e.g., schizophrenia, TIA, stroke, head trauma, epilepsy, major depressive disorder, bipolar disorder), absence of cardio- and cerebrovascular diseases, cancer, type I or II diabetes, non-progressive (e.g., post-traumatic) or reversible (e.g., metabolic type, by substance intoxication, by nutritional deficiencies) dementia, connective tissue diseases (e.g., systemic lupus erythematosus, Still disease), or respiratory or food allergies; no history of alcohol or drug abuse/addiction. 

The anthropometric measurements (i.e., weight and height) of each participant were detected, and, according to Quetelet’s formula (kg/m^2^), three subgroups were ranked based on BMI (normal-weight, overweight, and obese). The sample characteristics are summarized in Table 1. The whole sample included 164 normal-weight (108 females, BMI = 18.5–24.99, mean age = 21.63, SD = 2.47), 58 overweight (34 females, BMI = 25–29.99, mean age = 21.48, SD = 1.87), and 44 obese subjects (27 females, BMI > 30, mean age = 23.16, SD = 2.34). No difference between the three groups was found in the sex frequency (χ^2^(2) = 1.08, *p* = 0.58). Obese subjects scored higher on the age variable (F _2, 263_ = 8.38, *p* < 0.001, *η^2^* = 0.06).

### 2.2. Measures

Data were collected by using the following psychometric questionnaires: the Binge Eating Scale [54], the Emotional Overeating Questionnaire [55], and the Temperament and Character Inventory [50].

Binge Eating Scale (BES). This is a 16-item self-report questionnaire exploring behavioral (e.g., eating fast; eating in secret), cognitive, and emotional (e.g., loss of control, guilt) dimensions which typically characterize the BED (Cronbach’s α = 0.85). The score ranges from 0 to 46, with a higher score indicating more severe binge eating symptoms [54]. 

Emotional Overeating Questionnaire (EOQ). This 6-item self-report questionnaire has been devised for assessing overeating behaviors in response to six emotional states, namely, Anxiety, Sadness, Loneliness, Tiredness, Anger, and Happiness (Cronbach’s α = 0.85). The scoring set for each item consists of a 7-point Likert scale (0–6) measuring the frequency of overeating over the past 28 days. A higher score reflects more frequent overeating behaviors [55]. 

Temperament and Character Inventory (TCI). This is a 240-item self-report questionnaire assessing the four dimensions of temperament (i.e., Harm Avoidance, Novelty Seeking, Reward Dependence, and Persistence) and the three dimensions of character (i.e., Self-Directedness, Cooperativeness, and Self-Transcendence) according to Cloninger’s biopsychosocial model of personality (Cronbach’s α = 0.72). The scoring set for each item consists of a dichotomous “true/false” scale [49,50]. 

#### Ethics Statement

All participants gave prior written informed consent to participate in this study which was approved by the ethics committee of the University of Campania “Luigi Vanvitelli” and carried out according to the 1964 Declaration of Helsinki.

### 2.3. Statistical Analyses

The statistical analyses were performed based on a generalized linear model. Each variable’s score was standardized (z-score) in order to detect univariate outliers, and z-scores higher than |3| were removed. On the basis of skewness and kurtosis parameters, variables were additionally “normalized” by using square root (sqrt) or logarithmic (log_10_) transformations, if needed. 

To detect any differences between the three groups (normal-weight, overweight, obese) on the average self-report scores, descriptive statistics were improved by univariate analysis of variance (ANOVA). Pearson’s correlation analysis (*r*) was used to quantify the relationship between self-report scores, age, and BMI. Subsequently, to test the null hypothesis that the emotional (EOQ) and personality (TCI) dimensions did not determine statistically significant differences in overeating behavior (BES) between the three groups, an analysis of covariance (ANCOVA) was performed. More specifically, ANCOVA was applied assuming the BES score as a dependent variable and BMI, converted into a three-level ordinal variable (normal-weight, overweight, obese), as a fixed factor. Sex, age, EOQ, and TCI scores were simultaneously loaded in the model as covariates. Bonferroni’s correction for multiple comparisons was used as a post hoc test. The statistical significance was fixed at α < 0.05. The effect size was estimated by eta-squared (*η*^2^). Statistical analyses were performed using IBM SPSS Statistics (v. 20) and JASP packages.

## 3. Results

### 3.1. Descriptive Statistics

The mean BES, EOQ, and TCI scores for each group are shown in Table 2.

Binge Eating Scale (BES). A significant difference between groups in the BES score was observed (F _2, 263_ = 18.28, *p* < 0.001, *η^2^* = 0.12). More precisely, post hoc analyses showed that obese subjects scored higher compared to normal-weight subjects (mean difference: −1.24, SE = 0.21, t = −6.04, *p* < 0.001).

Emotional Overeating Questionnaire (EOQ). Normal-weight, overweight, and obese subjects significantly differed on Anger (F _2, 263_ = 10.64, *p* < 0.001, *η^2^* = 0.08), Anxiety (F _2, 263_ = 10.25, *p* < 0.001, *η^2^* = 0.07), Sadness (F _2, 263_ = 6.06, *p* < 0.05, *η^2^* = 0.05), Happiness (F _2, 263_ = 5.54, *p* < 0.01, *η^2^* = 0.04), Loneliness (F _2, 263_ = 4.89, *p* < 0.05, *η^2^* = 0.04), and total EOQ score (F _2, 263_ = 3.19, *p* < 0.05, *η^2^* = 0.02). Post hoc analyses showed that obese subjects scored significantly higher than normal-weight subjects on Anger (mean difference: −0.63, SE = 0.14, t = −4.59, *p* < 0.001), Anxiety (mean difference: −0.62, SE = 0.14, t = −4.51, *p* < 0.001), Sadness (mean difference: −0.15; SE = 0.06, t = −2.74, *p* < 0.05), Loneliness (mean difference: −0.34, SE = 0.14, t = −2.47, *p* < 0.05), and total EOQ score (mean difference: −0.69, SE = 0.28, t = −2.45, *p* < 0.05). Moreover, normal-weight subjects scored higher than overweight subjects on Happiness (mean difference: −0.36; SE = 0.12; t = −2.92, *p* < 0.05).

Temperament and Character Inventory (TCI). Normal-weight, overweight, and obese subjects significantly differed on Novelty Seeking (F _2, 263_ = 11.83, *p* < 0.001, *η^2^* = 0.09), Harm Avoidance (F _2, 263_ = 5.54, *p* < 0.01, *η^2^* = 0.04), and Cooperativeness (F _2, 263_ = 3.56, *p* < 0.05, *η^2^* = 0.03). Post hoc analyses showed that obese subjects scored lower on Novelty Seeking (mean difference: 1.65, SE = 0.36, t = 4.58, *p* < 0.001) and higher on Harm Avoidance (mean difference: −15.64, SE = 4.72, t = −3.31, *p* < 0.01) compared to normal-weight subjects. Although modestly, obese subjects also scored higher than overweight subjects on Cooperativeness (mean difference: −1.17, SE = 0.45, t = −2.59, *p* < 0.05). 

### 3.2. Pearson’s Correlation Analysis

The correlation matrix is reported in Table 3. A moderate correlation between BMI, BES (*r* = 0.37, *p* < 0.001), and Anxiety (*r* = 0.32, *p* < 0.001) emerged. Furthermore, BMI weakly correlated with Anger (*r* = 0.28, *p* < 0.001), Sadness (*r* = 0.20, *p* < 0.05), Harm Avoidance (*r* = 0.20, *p* < 0.01), total EOQ score (*r* = 0.15, *p* < 0.05), and Loneliness (*r* = 0.14, *p* < 0.05). In addition, BMI showed weak negative linear associations with both Novelty Seeking (*r* = −0.29, *p* < 0.001) and Happiness (*r* = −0.13, *p* < 0.05). Strong positive correlations between BES and Anger (*r* = 0.55, *p* < 0.001), total EOQ score (*r* = 0.50, *p* < 0.001), Sadness (*r* = 0.46, *p* < 0.001), Anxiety (*r* = 0.44, *p* < 0.001), Loneliness (*r* = 0.43, *p* < 0.001), and Harm Avoidance (*r* = 0.36, *p* < 0.001) were found. Conversely, BES showed a strong negative association with Self-Directedness (*r* = −0.45, *p* < 0.001). Weak correlations were also found between BES and Self-transcendence (*r* = 0.13, *p* < 0.05), Cooperativeness (*r* = −0.14, *p* < 0.05), and Persistence (*r* = −0.13, *p* < 0.05).

### 3.3. Analysis of Covariance (ANCOVA)

Heteroskedasticity was excluded by Levene’s test (F _2, 245_ = 0.41, *p* = 0.66). According to statistical tolerance, the total EOQ score was removed in order to mitigate collinearity effects. The results of the ANCOVA are shown in Table 4. Normal-weight, overweight, and obese subjects significantly differed on BES score regardless of age (F _1, 230_ = 2.32, *p* = 0.13, *η^2^* = 0.01) and gender (F _1, 230_ = 5.99, *p* < 0.05, *η^2^* = 0.02). As concerns emotional and personality variables, although Self-Directedness (F _1, 230_ = 11.25, *p* < 0.001, *η^2^* = 0.04), Sadness (F _1, 230_ = 6.77, *p* < 0.05, *η^2^* = 0.04), and Anger (F _1, 230_ = 10.45, *p* < 0.01, *η^2^* = 0.03) were found to be significant predictors of the BES vs. BMI relationship, the three groups significantly differed on BES score (F _2, 230_ = 7.55, *p* < 0.001, *η^2^* = 0.06) and also the net of the combined effect of personality and emotional variables. Moreover, a negligible contribution of Tiredness (F _1, 230_ = 4.94, *p* < 0.05, *η^2^* = 0.02) and Harm Avoidance (F _1, 230_ = 4.96, *p* < 0.05, *η^2^* = 0.02) was observed. The post hoc analysis showed statistically significant differences between normal-weight and obese subjects (mean difference: −0.67, SE = 0.19, t = −3.62, *p* < 0.01) and between overweight and obese subjects (mean difference: −0.52, SE = 0.20, t = −2.56, *p* < 0.05).

## 4. Discussion

The present study aimed at assessing the cumulative effect of EE and personality traits on overeating behavior in a young adult community sample stratified according to BMI. Our results show a dysfunctional pattern characterized by lower Self-Directedness combined with overeating in response to Sadness and Anger. Such a pattern significantly influenced the relationship between weight gain and overeating. This finding supports the presence of a cluster embracing negative emotions and weak abilities to modulate individual behavior [56]. 

Self-Directedness reflects general psychological functioning and internal organization [50]. By definition, Self-Directedness is a personality trait of self-determination, i.e., the ability to adapt one’s behavior to environmental demands in order to achieve personally chosen goals consistent with one’s own values. It is a metaphorical abstract concept describing the extent to which a person identifies the imaginal self as an integrated purposeful whole individual, rather than a disorganized set of reactive impulses. Interestingly, Self-Directedness has also been conceptually related to the locus of control. More precisely, some studies found that low Self-Directedness was associated with the external locus of control, while high Self-Directedness was associated with the internal locus of control [57]. Cloninger’s research showed that poor Self-Directedness is one of the main features of personality disorders [50,58].

In our study, however, OB-wed subjects significantly reported more binge episodes independently of the above-mentioned emotional-personological pattern and the net of the age and gender covariates entered in the generalized linear model. Taken together, these findings support the hypothesis that the emotional and personality domains might play only a marginal role in increasing obesity severity [11,25]. Considering this psychological pattern along a dimensional continuum ranging from healthy to psychopathological status, OB-wed subjects are likely below a clinical cut-off. In line with this claim, it has been suggested that OB-wed, unlike BED or other eating-related diseases, may not be strongly associated with abnormal personality characteristics [59]. 

Although personality/emotional dimensions might only marginally explain overeating behavior in OB-wed patients, their evaluation cannot be neglected in the clinical practice. Clinical studies consistently detected increased comorbidity between obesity, overeating, and personality disorders [60,61]. Furthermore, obese patients showed higher difficulties in persisting with long-term goals, body weight control, and functional adherence to weight loss programs [62,63]. Personality assessment is still important in the management and treatment of OB-wed patients. For instance, personality traits may predict the weight loss treatment outcome [61,64]. Moreover, certain TCI personality factors, such as high Reward Dependence and low Novelty Seeking, might be involved in successful weight control after behavioral weight loss treatment [11]. Furthermore, since drop-out phenomena are associated with the lowest probability of benefiting from treatment [65,66,67], studies designed to identify compliance and adherence to psychological therapy for obesity should also explore the factors leading to premature program interruption. It has been stated that personality traits would contribute to resistance to long-term modification [68]; further, they would be involved in functional adherence to treatment [46]. 

Obesity is a complex condition and a multifactorial psychological pattern that can contribute both as a risk and a maintenance factor [69,70]. Furthermore, obesity is strictly related to body schema arising from the integration of multisensory bodily input, and, when impaired, an inaccurate sensorimotor action representation is observed [71,72]. Body image representation, self-esteem, and specific executive subdomains [73] could be linked to behavioral alterations mediated by poorer Self-Directedness [74]. Similar to drug-addicted patients [75], obese subjects scored worse in decision-making tasks, showing a preference for immediately rewarding behavior despite the negative consequences that these choices produced in the long term [76,77,78,79]. 

The integrity of the dopamine system is needed to adaptively act across a variety of dynamic reward-seeking, motivational, and decision-making contexts [80]. Furthermore, it is involved in addiction behaviors [81]. The dopaminergic network presents a complex neural architecture beginning at the level of the midbrain (i.e., retrorubral area; substantia nigra pars compacta, SNc; ventral tegmental area, VTA) and extending in other regions including the hypothalamus, olfactory bulb, and retina [82]. The SNc receives excitatory afferent signals from the somatosensory and motor cortices and inhibitory inputs from the pars reticulata portion of the substantia nigra. Conversely, the VTA receives inputs from the hypothalamus, ventral pallidum, dorsal raphe, and prefrontal cortex [83]. The lateral habenula sends glutamatergic projections to the GABAergic Rostromedial Tegmental nucleus (RMTg), projecting inhibitory signals to the anterior VTA [84]. In other words, activity in the lateral habenula suppresses the activity of dopamine neurons.

Dopamine neurons projecting to the striatum allow assigning and learning the values of reward cues, formulating predictions, selecting appropriate actions, and enhancing motivational drives in response to rewards and threats. As observed in addiction patients, hyperactivation of VTA and SNc may determine compulsive conducts and behavioral inflexibility [85,86,87,88]. Interestingly, AgRP-expressing neurons in the hypothalamus arcuate nucleus are involved in the control of hunger, and, if hyper-stimulated, they could induce intense hunger and subsequent overeating behaviors [89]. Furthermore, it has been shown that the initial response to rewards, including food, evokes large dopamine responses in the nucleus accumbens core (NAC) [90,91]. Chronic dopamine depletion alters reward seeking when the effort required to pursue the reward is high [92]. In fact, NAC dopamine depletions suppress effortful reward seeking for a high-palatable food when a less palatable, low-effort option is also available [93]. 

Our study presents some criticisms. First, it does not allow drawing generalized conclusions about gender differences due to the imbalanced ratio of male/female in the BMI groups. However, the ANCOVA revealed a difference between BMI groups in the BES score regardless of a significant effect of gender. Therefore, our results might have a transversal nature. Second, EE and personality traits were evaluated by self-reported questionnaires which notoriously tend to overestimate the prevalence of psychopathologies [94]. Third, the results are restricted to university students enrolled in screening initiatives and do not provide any information on the larger number of obese individuals not seeking treatment or seeking help in non-clinical settings.

## 5. Conclusions

Our study detected the presence of a specific pattern characterized by lower Self-Directedness and higher Sadness/Anger influencing the frequency of binge episodes. We found that OB-wed subjects showed more binge episodes compared to overweight and normal-weight subjects, also removing the cumulative effect of emotional antecedents and personality traits. Although feeding behavior appeared to be triggered by Sadness, Anger, and a decreased ability to direct one’s own goals (i.e., lower Self-Directedness), our results show that obese subjects reported greater overeating behavior notwithstanding the effects of emotional and personological variables. Further investigations exploring the role of psychological features in relation to obesity are needed.

## Figures and Tables

**Table 1:** Anthropometric sample characteristics.

	Normal-Weight (n = 164)	Overweight (n = 58)	Obese (n = 44)
Gender (F/M)	108/56	34/24	27/17
Age, mean (SD)	21.63 (2.47)	21.48 (1.87)	23.16 (2.34)
Body mass index, mean (SD)	21.90 (1.69)	26.90 (1.39)	35.48 (4.56)

**Table 2:** Mean scores (SDs) for each scale.

	Normal-Weight (n = 164)	Overweight (n = 58)	Obese (n = 44)	Sig.
*Binge Eating Scale*	7.62 (6.35)	9.79 (7.44)	15.82 (9.88)	***
*Emotional Overeating Questionnaire*				
Sadness	1.06 (1.35)	1.14 (1.52)	1.95 (1.91)	*
Anxiety	0.94 (1.32)	1.41 (2.03)	2.38 (2.11)	***
Loneliness	1.15 (1.32)	1.03 (1.56)	2.19 (2.22)	*
Tiredness	0.99 (1.56)	0.83 (1.52)	1.14 (1.83)	
Anger	0.85 (1.38)	1.21 (1.64)	2.14 (2.08)	***
Happiness	1.77 (1.93)	1 (1.54)	1.1 (1.53)	**
Total Score	6.68 (5.89)	6.62 (7.65)	10.41 (8.92)	*
*Temperament and Character Inventory*				
Harm Avoidance	65.79 (28.27)	70.38 (27.44)	81.43 (21.64)	**
Novelty Seeking	62.36 (28.48)	51.21 (28.54)	39.57 (22.82)	***
Reward Dependence	43.17 (26.98)	35.41 (22.49)	44.24 (23.12)	
Cooperativeness	42.43 (23.89)	34.62 (21.9)	48.24 (24.6)	*
Persistence	29.26 (27.34)	30.83 (26.07)	28.52 (20.88)	
Self-Directedness	36.43 (26.73)	32.31 (25)	34.71 (28.06)	
Self-Transcendence	27.57 (24.81)	31.45 (25.8)	30.86 (25.42)	

Note: ANOVAs were significant at * *p* < 0.05, ** *p* < 0.01, *** *p* < 0.001.

**Table 3:** Correlation matrix.

	Age	BMI	BES	EOQ-Sad	EOQ-Anx	EOQ-Lon	EOQ-Tir	EOQ-Ang	EOQ-Hap	EOQ-Tot	TCI-HA	TCI-NS	TCI-RD	TCI-C	TCI-P	TCI-SD	TCI-ST
Age	—																
BMI	0.17 **	—															
BES	0.05	0.37 ***	—														
EOQ-Sad	0.08	0.20 *	0.46 ***	—													
EOQ-Anx	0.09	0.32 ***	0.44 ***	0.51 ***	—												
EOQ-Lon	0.03	0.14 *	0.43 ***	0.48 ***	0.36 ***	—											
EOQ-Tir	−0.02	0.04	0.10	0.36 ***	0.35 ***	0.25 ***	—										
EOQ-Ang	0.14 *	0.28 ***	0.55 ***	0.55 ***	0.57 ***	0.45 ***	0.38 ***	—									
EOQ-Hap	−0.03	−0.13 *	0.15 *	0.09	0.23 ***	0.25 ***	0.46 ***	0.28 ***	—								
EOQ-Tot	0.03	0.15 *	0.50 ***	0.73 ***	0.70 ***	0.67 ***	0.63 ***	0.74 ***	0.64 ***	—							
TCI-HA	0.16 *	0.20 **	0.36 ***	0.02	0.26 ***	0.33 ***	0.13*	0.29 ***	0.20 **	0.36 ***	—						
TCI-NS	−0.07	−0.29 ***	0.01	−0.12	−0.12	0.05	−0.23 ***	−0.10	0.11	−0.05	−0.32 ***	—					
TCI-RD	0.13 *	0.01	0.11	−0.23 **	0.07	0.05	0.06	0.11	0.23 ***	0.13 *	0.12	0.08	—				
TCI-C	0.13 *	−0.01	−0.14 *	−0.20 *	0.11	−0.19 **	0.08	0.01	0.05	−0.02	−0.01	−0.10	0.43 ***	—			
TCI-P	−0.12	0.07	−0.13 *	0.10	0.09	−0.10	0.03	−0.00	−0.07	−0.05	−0.19 **	−0.13 *	0.06	0.04	—		
TCI-SD	0.08	−0.07	−0.45 ***	−0.27 ***	−0.21 ***	−0.34 ***	−0.08	−0.32 ***	−0.10	−0.30 ***	−0.35 ***	−0.06	0.02	0.40 ***	0.26 ***	—	
TCI-ST	0.05	0.01	0.13 *	0.18 *	0.16*	0.05	0.23 ***	0.16 **	0.20 **	0.22 ***	−0.08	−0.04	0.18 **	0.19 **	0.15 *	−0.07	—

BMI: body mass index; BES: Binge Eating Scale; EOQ: Emotional Overeating Questionnaire; EOQ-Sad: Sadness; EOQ-Anx: Anxiety; EOQ-Lon: Loneliness; EOQ-Tir: Tiredness; EOQ-Ang: Anger; EOQ-Hap: Happiness; EOQ-Tot: total score; TCI-HA: Harm Avoidance; TCI-NS: Novelty Seeking; TCI-RD: Reward Dependence; TCI-C: Cooperativeness; TCI-P: Persistence; TCI-SD: Self-Directedness; TCI-ST: Self-Transcendence. Note: * *p* < 0.05, ** *p* < 0.01, *** *p* < 0.001.

**Table 4:** Analysis of covariance.

	Sum of Squares	Mean Square	F	*p*	*η^2^*
BMI * BES	10.99	5.50	7.55	***	0.06
Sex	5.03	5.03	5.99	*	0.02
Age	1.94	1.94	2.32		
EOQ-Sad	4.96	4.96	6.77	***	0.04
EOQ-Anx	1.18	1.18	1.41		
EOQ-Lon	0.01	0.01	0.01		
EOQ-Tir	4.15	4.15	4.94	*	0.02
EOQ-Ang	8.77	8.77	10.45	***	0.03
EOQ-Hap	0.01	0.01	0.01		
TCI-HA	4.16	4.16	4.96	*	0.02
TCI-NS	1.47	1.47	1.75		
TCI-RD	2.53	2.53	3.01		
TCI-C	2.98	2.98	3.55		
TCI-P	0.13	0.13	0.16		
TCI-SD	9.44	9.44	11.25	***	0.04
TCI-ST	1.09	1.09	1.30		

BMI: body mass index; BES: Binge Eating Scale; EOQ: Emotional Overeating Questionnaire; EOQ-Sad: Sadness; EOQ-Anx: Anxiety; EOQ-Lon: Loneliness; EOQ-Tir: Tiredness; EOQ-Ang: Anger; EOQ-Hap: Happiness; TCI-HA: Harm Avoidance; TCI-NS: Novelty Seeking; TCI-RD: Reward Dependence; TCI-C: Cooperativeness; TCI-P: Persistence; TCI-SD: Self-Directedness; TCI-ST: Self-Transcendence. Note: * *p* < 0.05, *** *p* < 0.001.

## Data Availability

The data presented in this study are available on request from the corresponding author.
